# Supervised Cycling Training Improves Erythrocyte Rheology in Individuals With Peripheral Arterial Disease

**DOI:** 10.3389/fphys.2021.792398

**Published:** 2022-01-05

**Authors:** Chih-Chin Hsu, Yu-Ting Lin, Tieh-Cheng Fu, Shu-Chun Huang, Cheng-Hsien Lin, Jong-Shyan Wang

**Affiliations:** ^1^Department of Physical Medicine and Rehabilitation, Keelung Chang Gung Memorial Hospital, Keelung, Taiwan; ^2^School of Medicine, College of Medicine, Chang Gung University, Taoyuan City, Taiwan; ^3^Healthy Aging Research Center, College of Medicine, Graduate Institute of Rehabilitation Science, Chang Gung University, Taoyuan City, Taiwan; ^4^Department of Physical Medicine and Rehabilitation, Linkou Chang Gung Memorial Hospital, Taoyuan City, Taiwan; ^5^Department of Physical Medicine and Rehabilitation, New Taipei Municipal Tucheng Hospital, New Taipei City, Taiwan; ^6^Research Center for Chinese Herbal Medicine, College of Human Ecology, Chang Gung University of Science and Technology, Taoyuan City, Taiwan

**Keywords:** aerobic exercise, erythrocyte deformability, hemorheology, erythrocyte osmotic fragility, peripheral arterial disease

## Abstract

Peripheral arterial disease (PAD) results in insufficient flow to lower extremities. Aerobic exercise provides health benefits for individuals with PAD, but basic science behind it is still debated. Twenty-one PAD patients aged about 70 years with female/male as 7/14 were recruited. Among them, 11 were randomized to have supervised cycling training (SCT) and 10 to receive general healthcare (GHC) as controls. SCT participants completed 36 sessions of SCT at the first ventilation threshold within 12 weeks and the controls received GHC for 12 weeks. Ankle-brachial index (ABI), 6-min walk test (6MWT), peak oxygen consumption ($\dot{\text{V}}$O_2peak_), minute ventilation ($\dot{\text{V}}$_E_), minute carbon dioxide production ($\dot{\text{V}}$CO_2_), erythrocyte rheology, including the maximal elongation index (EI_max_) and shear stress at 50% of maximal elongation (SS_1/2_), and the Short Form-36 (SF-36) questionnaire for quality of life (QoL) were assessed before and 12 weeks after initial visit. SCT significantly decreased the SS_1/2_ as well as SS_1/2_ to EI_max_ ratio (SS_1/2_/EI_max_) and increased the erythrocyte osmolality in the hypertonic region as well as the area under EI-osmolality curve. The supervised exercise-induced improvement of erythrocyte deformability could contribute to the increased peripheral tissue O_2_ delivery and was possibly related with increased $\dot{\text{V}}$O_2peak_. The physiological benefit was associated with significantly increased ABI, 6-min walking distance, cardiorespiratory fitness, and SF-36 score. However, no significant changes in aerobic capacity and erythrocyte rheological properties were observed after 12-week of GHC. In conclusion, SCT improves aerobic capacity by enhancing erythrocyte membrane deformability and consequently promotes QoL in PAD patients.

## Introduction

Peripheral arterial disease (PAD), a progressive atherosclerotic disease, is characterized by arterial stenosis and consequently reduces ordinary physical activities (Kithcart and Beckman, 2018). A drop in pressure and turbulence are observed when blood flows across the stenosis (Hiatt et al., 2015). The pathophysiology prevents sufficient flow in the lower extremity and further results in reduced ankle-brachial index (ABI), mis-matched oxygen supply–demand, and high oxidative stress (Zalawadiya et al., 2012; Desormais et al., 2014; Velioglu and Yuksel, 2019) in PAD individuals. Endothelial dysfunction, resulted from the oxidative stress-induced generation of superoxide anion, impairs vasodilatation adaptations during exercise (Hiatt et al., 2015). Therefore, promoting physical activities for PAD patients become a great challenge in modern medicine.

Decreased exercise capacity associated with PAD individuals results in reduced functional independence and further impairs quality of life (QoL) (Treat-Jacobson et al., 2019a,b). Erythrocytes have emerged as the core determining factor of blood rheology, whereas erythrocyte rheological dysfunction results in circulatory disorders (Kensey, 2003; Barshtein et al., 2007). A significant correlation between the progressive deterioration of peripheral circulatory disturbances and the susceptibility of erythrocyte membrane lipids to oxidative stress has been observed (Cho et al., 2014). Increased erythrocyte deformability has been identified to be associated with increased exercise performance in athletes (Guezennec et al., 1989). Pentoxifylline, a xanthine derivative, has been shown to improve walking distance in PAD patients (Accetto, 1982; Angelkort et al., 1985) by improving erythrocyte deformability (Ouriel, 2001). Despite increasing cardiopulmonary and muscular fitness (Treat-Jacobson et al., 2019a,b), aerobic exercise training effects on erythrocyte hemorheological properties and the ability to deliver O_2_ to tissues in PAD patients remain unclarified.

Erythrocyte rheological properties affect blood viscoelasticity and consequently regulate vascular resistance to blood flow shear stress. Moreover, osmolality-mediated erythrocyte deformability plays a meaningful role in hemorheology during exercise (Baskurt and Meiselman, 2003). In our recent study of healthy sedentary males, cycling training at 60% of $\dot{\text{V}}$O_2_ reserve ($\dot{\text{V}}$O_2_R) improved the erythrocyte membrane stability and osmotic deformability (Huang et al., 2019). However, aerobic exercise effects on erythrocyte membrane stability and osmotic deformability in PAD patients have not yet been established. To understand how aerobic exercise affects erythrocyte membrane stability as well as osmotic deformability and aerobic capacity in PAD, the present study aimed to establish an effective exercise regimen to improve erythrocyte rheological functionality and to enhance cardiorespiratory fitness in individuals with PAD.

## Materials and Methods

### Subjects

This study was approved by the Institutional Review Board of a tertiary care hospital. The ClinicalTrials.gov number is NCT03965520. Individuals with lower-extremity PAD were surveyed for the intervention from April 1, 2018 to December 31, 2019 at our hospital. Individuals aged >20 years with PAD >2 weeks, ankle-brachial index (ABI) ≤ 0.9, and capable of participating in active exercise were included. However, individuals with unstable angina, systolic blood pressure (SBP) at rest >200 mmHg or diastolic blood pressure (DBP) >110 mmHg, orthostatic blood pressure decrease >20 mmHg with symptoms, severe aortic stenosis, acute discomfort or fever, uncontrolled atrial or ventricular dysrhythmia, and uncontrolled sinus rhythm tachycardia (>120/min) decompensated heart failure, third-degree atrioventricular block, acute pericarditis or myocarditis, recent embolism, thrombophlebitis, ST segment displacement >2 mm at rest, and uncontrolled diabetes were not candidates for present work. All eligible PAD individuals provided informed consent after the experimental procedures were explained. Then, a computer-generated, concealed allocation schedule randomly assigned participants to receive SCT or general healthcare (GHC). Lipoprotein profiles, including total cholesterol, high-density lipoprotein (HDL), low-density lipoprotein (LDL), and triglyceride levels, were also determined. Data were collected by an assessor with blinding to group before randomization and after completing the 12-week observational period.

### ABI Measurement

Doppler measurements were performed according to the American Heart Association guidelines for ABI measurement (Guirguis-Blake et al., 2018; Misra et al., 2019). A digital Doppler spectrum analysis device equipped with an 8-MHz probe (Dopplex DMX; Huntleigh Healthcare, United States) was used to measure individual systolic pressure. An appropriately sized pneumatic cuff was applied to the arm and the same side ankle joint. Cuff was inflated to supra-systolic pressure and was deflated slowly until Doppler flow signals in brachial (Br) and dorsalis pedis (DP) arterial pulses were detected. The process was performed for both legs, and values were calculated for each lower limb separately as systolic DP pressure/systolic Br pressure.

### Cardiopulmonary Exercise Test

All participants were instructed to fast for at least 8 h and to refrain from exercise for at least 24 h before the test. All participants arrived at the testing center at 9:00 a.m. to eliminate diurnal effects. They underwent an incremental exercise test 2 days before and 2 days after 12-week of interventions on a bicycle ergometer (Ergoselect 150P, ergoline GmbH, Bitz, Germany) at an increased work-rate of 10 W/min. During Cardiopulmonary exercise test (CPET), heart rate (HR) and brachial blood pressure were measured by an automatic blood pressure monitoring system (Tango, SunTech Medical Inc., Morrisville, NC, United States), and arterial oxygen saturation was tracked by a pulse-oximeter (model 9500, Nonin Onyx, Plymouth, MN, United States) until the stop conditions described previously. (Fu et al., 2013; Huang et al., 2019; Hsu et al., 2021).

Minute ventilation ($\dot{\text{V}}$_E_), $\dot{\text{V}}$O_2_, and minute carbon dioxide production ($\dot{\text{V}}$CO_2_) were measured by a breath-by-breath basis using a computer-based system (MasterScreen CPX, CareFusion Corp., Hoechberg, Germany). Ventilation threshold (VT) and $\dot{\text{V}}$O_2peak_ were defined as our previous work based on the ACSM guideline for exercise testing (Pescatello et al., 2014). $\dot{\text{V}}$_E_ and $\dot{\text{V}}$CO_2_ responses, acquired from the initiation of exercise to the end of CPET, were used to calculate the $\dot{\text{V}}$_E_-$\dot{\text{V}}$CO_2_ slope, using least-squares linear regression (y = m · x + b, m = slope) (Arena et al., 2007), Additionally, walking distances obtained at pain-free (initial) and intolerable leg pain (terminal) status during the 6-min walk test (6MWT) was recorded to determine exercise endurance (Montgomery and Gardner, 1998).

### SCT Protocol

All participants received GHC were instructed to undergo symptom-limited and self-paced walking exercise in the community as well as regular pharmacological therapy at our medical clinic. Participants without additional SCT were treated as the GHC group. In addition to the daily activity, the SCT group also performed 36 sessions (three sessions per week for 12 weeks) of in-hospital supervised exercise training on a bicycle ergometer. The SCT protocol comprised a warm-up at 30% of $\dot{\text{V}}$O_2peak_ (watt power corresponding to 30% $\dot{\text{V}}$O_2peak_) for 3 min, followed by continuous watt power corresponding to the first VT for 30 min as our previous work (Fu et al., 2013), then a cool-down at 30% of $\dot{\text{V}}$O_2peak_ (watt power corresponding to 30% $\dot{\text{V}}$O_2peak_) for 3 min. The first VT is a marker of intensity that can be observed in a person’s breathing at a point where lactate begins to accumulate in the blood. Aerobic exercise training at first VT intensity improved cardiopulmonary and functional capacities of patients with circulatory disorders (Pymer et al., 2020). We increased exercise intensity during the exercise training by approximately 10% HR reserve every 2 weeks as the participant could tolerate (Hsu et al., 2021). The training was terminated when participants had symptoms/signs suggested by the ACSM Guidelines (Pescatello et al., 2014). The compliance rate for the SCT and GHC groups was 100% for both.

### Measurement of Erythrocyte Rheological Properties

At rest and immediately after the graded exercise test, a 20-mL-blood sample was collected from the antecubital vein *via* clean venipuncture (20-gauge needle) under controlled venous stasis at 40 mmHg. A blood sample of 10 ml was added to a tube with ethylenediaminetetraacetic acid (final concentration, 4 mM) for measuring erythrocyte rheological characteristics, and another blood sample of 10 ml was added to a tube containing sodium citrate concentration 3.8 g/dl with a volume ratio of 9 to 1 for evaluating membrane stability and osmotic deformability of erythrocytes. Blood cells were counted by using a cell counter (Sysmax SF-3000, GMI Inc., Ramsey, MN, United States). These tests were performed immediately after blood sampling (Huang et al., 2019).

Sodium citrate was a common anticoagulant for blood cell function test. Like EDTA, citrate acted by removing calcium from the blood. Unlike EDTA, it is reversible—so calcium can be added back to study blood cell function under controlled conditions. Citrated plasma was also used to measure hemostatic-relevant factors related to peripheral arterial disorder (Kennedy et al., 2021).

### Erythrocyte Membrane Stability Test

A 25-μL blood sample with sodium citrate was diluted in 5 ml isotonic (osmolality = 290–300 mOsm/kg, pH 7.3) polyvinylpyrrolidone (PVP) solution (Sigma-Aldrich, St. Louis, MO, United States). Then, 1,000 μl of the PVP-diluted blood sample was added to the sheared sample system in a laser-assisted optical rotational red cell analyzer (LoRRca, RR Mechatronics, Hoorn, Netherlands) at 37°C for elongation index (EI) measurements at various fluid shear stresses by laser diffraction analysis (Simmonds et al., 2014; Huang et al., 2019).

A laser beam directly passed through the sheared sample, and the diffraction pattern produced by the deformed erythrocytes was analyzed by using a computer. The EI was calculated according to the geometry of the elliptical diffraction pattern: EI = (L − W)/(L + W), where L and W represent the length and width of the diffraction pattern, respectively. The shear stress at 50% of maximal elongation was defined as semi-maximal shear stress (SS_1/2_). Hence, an increase in SS_1/2_ or ratio of SS_1/2_ to EI_max_ represents a decrease in erythrocyte deformability (Simmonds et al., 2014; Huang et al., 2019).

The erythrocyte membrane stability test included the initial deformability curve test at basal status and the secondary deformability curve test following the 50 Pa of shear stress for 30 min. These deformability curves present the shear stress-EI curves for 10 consecutive shear stresses: 0.3, 0.54, 0.96, 1.73, 3.1, 5.56, 9.97, 17.88, 32.07, and 57.50 Pa. The experimental results provide a model that may be used to predict the change in erythrocyte deformability following exposure to a pathological shear stress, which resembles the flow condition in stenotic arteries.

### Erythrocyte Osmotic Deformability Test

The osmotic gradient ektacytometric measurements (Heo et al., 2015; Nemeth et al., 2015; Huang et al., 2019) were obtained using the “osmoscan function” of the above optical rotational red cell analyzer device (LoRRca). The device generated a constant shear stress of 30 Pa while continuously aspirating the sample into the measurement site and while changing the osmolarity of the medium by using gradual mixtures of PVP solutions of 0 and 700 mOsmol/kg; therefore, the EI was continuously registered. The parameters measured and calculated by the device (all at shear stress of 30 Pa) were as follows (Heo et al., 2015; Nemeth et al., 2015; Huang et al., 2019):

O_max_ (osmolality at EI_max_);
O_hyper_ (osmolality in the hypertonic region corresponding to 50% EI_max_);
EI_max_;
EI_hyper_ (EI in O_hyper_); and
Area under the individual EI-osmolarity curve (AUC).

All the above parameters before and after interventions were determined by the EI-osmolarity curve (see Supplementary Figure S1).

### Health-Related Quality of Life

Health-related QoL was measured by the Medical Outcomes Study 36-item Short Form (SF-36). The SF-36 questionnaire is a generic measure and can help differentiate QoL issues related to co-morbidities from those related to circulatory diseases (Maksimovic et al., 2014). We measured the physical component (PCS) and mental component (MCS) scores of the SF-36 questionnaire.

### Statistical Analysis

Data are expressed as mean ± SD and were analyzed by StatView (StatView 5.0; SAS Institute Inc., Cary, NC, United States). Experimental results were analyzed by 2 (groups) × 2 (time sample points; i.e., pre- and post-intervention) repeated-measure ANOVA with Bonferroni’s post-hoc test to compare cardiopulmonary fitness and erythrocyte rheological properties at the beginning of the study and after 12 weeks in the SCT and GHC groups. The criterion for significance was *p* < 0.05.

## Results

### Clinical Characteristics

**Figure 1:**
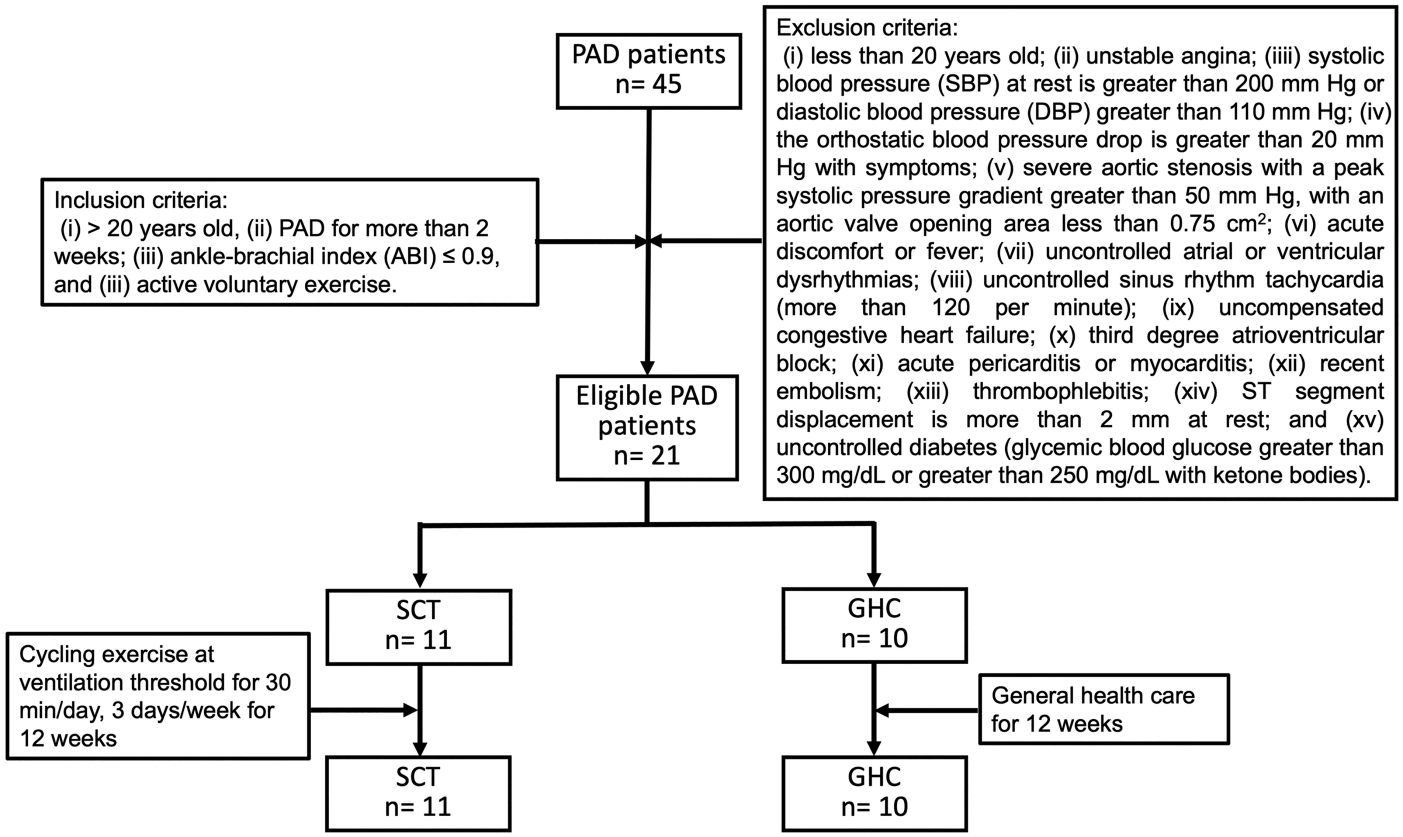
Flowchart of participants with peripheral arterial disease (PAD) during follow-up. Recruited PAD patients were divided into two groups: supervised cycling training (SCT) and general healthcare (GHC). The SCT group performed supervised hospital-based training on a bicycle ergometer (i.e., cycling exercise at ventilation threshold for 30 min/day, 3 days/week for 12 weeks). The GHC group had only general healthcare for 12 weeks, as instructed by their physician.

Initially, 45 individuals with PAD were surveyed according to our inclusion criteria. Among them, 24 were excluded based on our exclusion criteria, and 21 PAD were included in the study. All the participants underwent physical activities as recommended and received regular pharmacological therapy at our medical clinic. Among them, 11 were randomly allocated to the SCT, and 10 to the GHC groups (Figure 1). No adverse hemodynamic or hemorheological event occurred in the two groups throughout the investigation period. Moreover, baseline information was not significantly different between the two groups (Table 1).

**Table 1:** Pre- and post-intervention demographic and clinical characteristics of participants with peripheral arterial disease undergoing supervised cycling training (SCT) or general healthcare (GHC).

	SCT (n = 11)		GHC (n = 10)	
	Pre	Post	Pre	Post
**Anthropometrics/clinical characteristics**				
Sex (M/F)	7/4	7/4	7/3	7/3
Age (years)	70.3 ± 3.2	–	69.3 ± 3.4	–
Height (cm)	164.3 ± 4.2	–	163.5 ± 4.5	–
Weight (kg)	68.5 ± 3.1	68.3 ± 3.6	68.2 ± 3.4	68.7 ± 3.6
Heart rate (bpm)	79 ± 3	78 ± 4	81 ± 4	82 ± 4
Systolic blood pressure (mmHg)	141 ± 5	138 ± 6	142 ± 5	143 ± 6
Diastolic blood pressure (mmHg)	84 ± 4	81 ± 4	86 ± 4	85 ± 5
**Lipoprotein profile**				
T-CHO (mg/dL)	188 ± 46	185 ± 42	182 ± 33	186 ± 47
LDL (mg/dL)	116 ± 38	116 ± 32	113 ± 30	119 ± 63
HDL (mg/dL)	40.7 ± 11	43.7 ± 16	41.2 ± 14	44.2 ± 13
TG (mg/dL)	149 ± 83	146 ± 61	145 ± 83	146 ± 92
**Comorbidity, n**				
CAD	5	–	4	–
Hyperlipidemia	6	–	6	–
Hypertension	5	–	5	–
Diabetes mellitus	7	–	7	–
**Medicines, n**
Anti-platelet	5	5	5	5
Statins	6	6	6	6
β-blockers	8	8	7	7
ACEI/ARB	7	7	6	6
CCB	3	3	3	3
Sulfonylurea	6	6	5	5
DDP-4 inhibitor	3	3	3	3
SGLT2 inhibitor	2	2	2	2

*Values are mean ± SD or n. ACEI: angiotensin-converting enzyme inhibitor; ARB: angiotensin II receptor blocker; CAD: coronary artery disease; CCB: calcium channel blocker; DPP-4: dipeptidyl peptidase 4; F: female; HDL: high-density lipoprotein; L: left; LDL: low-density lipoprotein; M: male; R: right; SGLT2: sodium-glucose transport protein 2; T-CHO: total cholesterol; and TG: triglyceride*.

### Physiological Adaptations to Exercise

For cardiorespiratory fitness, 12-week of SCT significantly increased the peak work-rate, HR, $\dot{\text{V}}$_E_, and $\dot{\text{V}}$CO_2_ at (*p* < 0.05). Moreover, significantly lowered $\dot{\text{V}}$_E_-$\dot{\text{V}}$CO_2_ slope (from 36.4 to 33.1, *p* < 0.05), higher $\dot{\text{V}}$O_2peak_ (from 14.7 ml/min/kg to 18.6 ml/min/kg, *p* < 0.05), and greater 6MWT distances (initial distance from 222.4 m to 329.2 m; terminal point from 367.2 m to 456.0 m, *p* < 0.05) were also observed in PAD individuals after 12 week of SCT. The exercise intervention also significantly increased ABI values of the right (R) and left (L) lower extremities (R ratio from 0.83 to 0.91; L ratio from 0.84 to 0.92, *p* < 0.05). Conversely, cardiorespiratory fitness, walking distance, and lower-extremity circulation in the GHC participants did not show significant change during the follow-up (Table 2).

**Table 2:** Pre- and post-intervention effects on lower-extremity pulse, cardiorespiratory fitness, and health-related quality of life in the SCT and GHC participants.

	SCT		GHC	
	Pre	Post	Pre	Post
**Peak exercise performance**				
Work-rate (watt)	58.4 ± 5.5	78.2 ± 6.5^*^,^+^	56.1 ± 4.2	59.3 ± 5.0
Heart rate (bpm)	116 ± 5	131 ± 4^*^,^+^	119 ± 6	122 ± 5
$\dot{\text{V}}$_E_ (L/min/kg)	34.8 ± 3.4	48.5 ± 4.2^*^,^+^	36.3 ± 4.5	39.5 ± 4.3
$\dot{\text{V}}$O_2_ (mL/min/kg)	14.7 ± 2.7	18.6 ± 2.6^*^,^+^	14.4 ± 2.4	14.8 ± 2.6
$\dot{\text{V}}$CO_2_ (mL/min/kg)	15.4 ± 2.8	21.5 ± 2.9^*^,^+^	15.7 ± 2.3	15.9 ± 2.5
$\dot{\text{V}}$_E_-$\dot{\text{V}}$CO_2_ slope	36.4 ± 2.4	33.1 ± 1.8^*^,^+^	36.2 ± 3.9	36.5 ± 2.7
**6-min walk test**				
Initial distance (m)	222.4 ± 23.5	329.2 ± 34.6^*^,^+^	226.3 ± 31.4	241.5 ± 33.6
Terminal distance (m)	367.2 ± 37.7	456.0 ± 46.7^*^,^+^	372.6 ± 36.4	376.6 ± 41.6
**Ankle-brachial index**				
R	0.83 ± 0.03	0.91 ± 0.04^*^,^+^	0.85 ± 0.04	0.86 ± 0.05
L	0.84 ± 0.03	0.92 ± 0.04^*^,^+^	0.84 ± 0.04	0.83 ± 0.05
**Medical outcomes study 36-item short form**				
PCS	38.4 ± 4.7	44.6 ± 4.5^*^,^+^	39.3 ± 4.6	41.4 ± 5.4
MCS	36.3 ± 4.6	43.6 ± 4.1^*^,^+^	36.6 ± 5.5	40.1 ± 5.3

*Values are mean ± SD. GHC: general healthcare; MCS: mental component score; L: left; PCS: physical component score; Post: after intervention; Pre: before intervention; R: right; SCT: supervised cycling training;* $\dot{\text{V}}$*_E_: minute ventilation;* $\dot{\text{V}}$*CO_2_: minute CO_2_ production; and* $\dot{\text{V}}$*O_2_: O_2_ consumption*.

* *p < 0.05: pre* vs. *post*;

+ *p < 0.05: SCT* vs. *GHC.*

### Health-Related QoL

The SCT for 12 weeks significantly increased the scores of the PCS (scores from 38.4 to 44.6, *p* < 0.05) and MCS (from 36.3 to 43.6, *p* < 0.05) of the SF-36. However, the two component scores remained unchanged with GHC alone (Table 2).

### Blood Erythrocyte Characteristics

We found no significant changes in erythrocyte count, hemoglobin level, hematocrit, mean corpuscular volume, mean corpuscular hemoglobin level, and erythrocyte distribution of width-standard deviation or -coefficient of variance after 12 weeks of SCT or GHC (Table 3).

**Table 3:** Pre- and post-intervention effects on blood erythrocyte characteristics and osmotic deformability in of SCT and GHC participants.

	SCT		GHC	
	Pre	Post	Pre	Post
**Erythrocyte characteristics**				
RBC (10^6^/μL)	4.51 ± 0.77	4.49 ± 0.42	4.59 ± 0.79	4.54 ± 0.84
Hemoglobin (dL)	13.3 ± 2.0	13.6 ± 1.3	13.5 ± 2.1	13.4 ± 2.3
Hematocrit (%)	45.0 ± 3.3	45.4 ± 3.9	45.1 ± 3.2	45.0 ± 3.3
MCV (fL)	92.0 ± 4.9	93.5 ± 3.6	92.0 ± 4.9	90.4 ± 4.9
MCH (pg)	29.7 ± 1.5	30.3 ± 1.3	29.6 ± 1.6	29.3 ± 1.8
MCHC (g/dL)	32.3 ± 1.0	32.1 ± 0.8	32.5 ± 1.2	32.4 ± 1.0
RDW-SD (fL)	43.2 ± 2.6	43.5 ± 3.3	43.2 ± 3.1	43.9 ± 4.1
RDW-CV (%)	13.2 ± 0.9	13.5 ± 0.8	13.4 ± 1.1	13.7 ± 2.3
**Erythrocyte osmotic deformability**				
O_max_ (mOsm/kg.H_2_O)	297 ± 8	302 ± 15	298 ± 13	301 ± 18
O_min_ (mOsm/kg.H_2_O)	142 ± 6	147 ± 8	143 ± 7	145 ± 6
O_hyper_ (mOsm/kg.H_2_O)	452 ± 8	472 ± 7*	460 ± 11	465 ± 12
EI_max_	0.585 ± 0.038	0.597 ± 0.038	0.594 ± 0.039	0.596 ± 0.043
EI_min_	0.119 ± 0.006	0.129 ± 0.006	0.125 ± 0.012	0.126 ± 0.011
EI_hyper_	0.292 ± 0.012	0.298 ± 0.008	0.293 ± 0.007	0.295 ± 0.012
AUC	165 ± 2	174 ± 3*	167 ± 4	170 ± 6

*Values are mean ± SD. AUC: area under the individual elongation index-osmolarity curve; EI_hyper_, EI in osmolality in the hypertonic region corresponding to 50% of the maximal elongation index; EI_max_, the maximal elongation index; EI_min_, the minimal elongation index; GHC, general health care; MCV, mean corpuscular volume; MCH, mean corpuscular hemoglobin; MCHC, mean corpuscular hemoglobin concentration; O_max_, osmolality at EI_max_; O_min_, osmolality at EI_min_; O_hyper_, osmolality in the hypertonic region corresponding to 50% EI_max_; Post, after intervention; Pre, before intervention; RBC, red blood cell; RDW-SD or –CV, erythrocyte distribution width-standard deviation or -coefficient of variation; and SCT, supervised cycling training*.

* *p < 0.05: pre* vs. *post*.

### Erythrocyte Deformability and Membrane Stability

**Figure 2:**
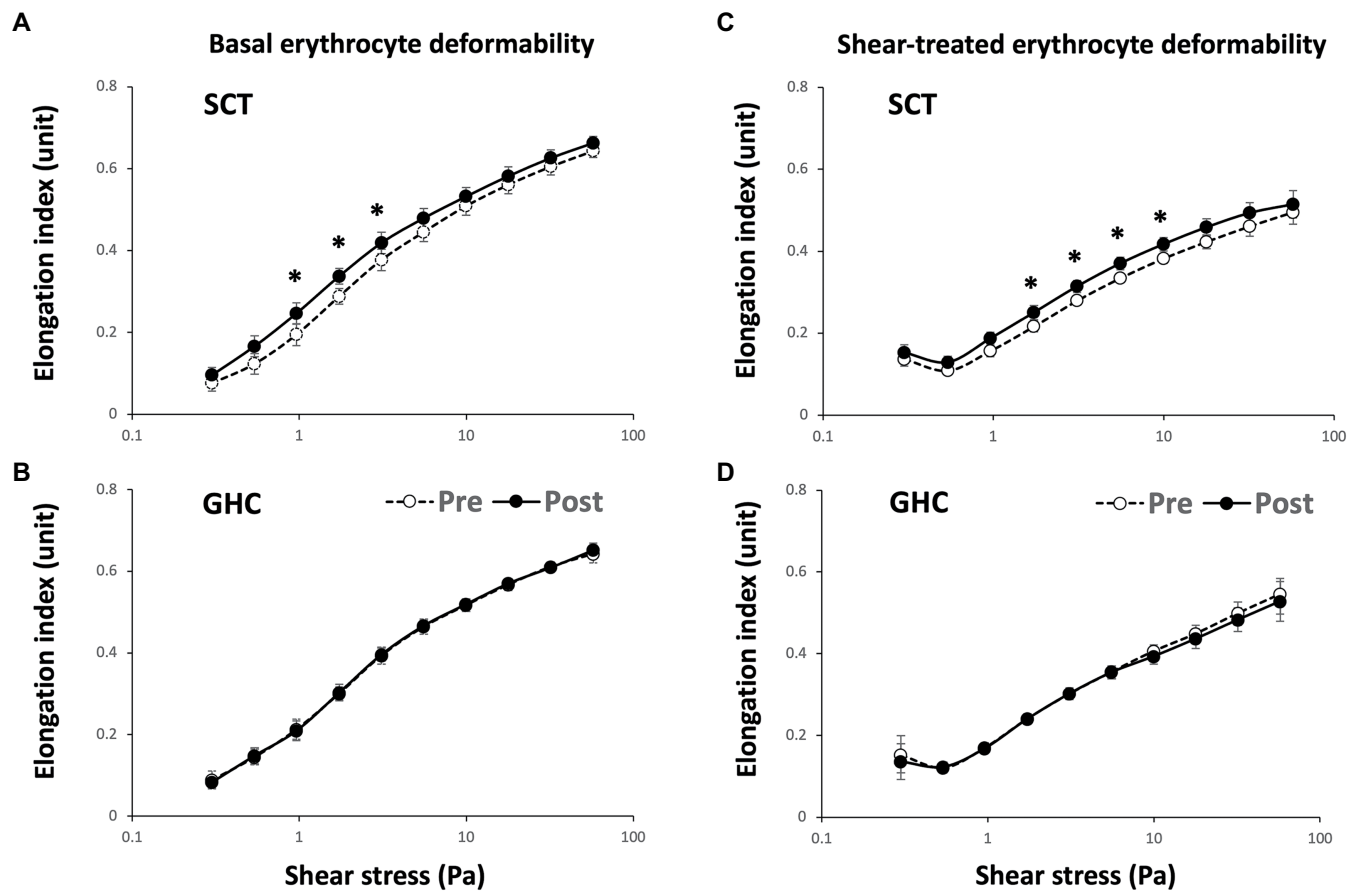
Effect of SCT and GHC on erythrocyte membrane stability in participants with PAD. The erythrocyte membrane stability test included the initial deformability curve test at basal status [**(A)** and **(B)** basal erythrocyte deformability] and the secondary deformability curve test followed the 50-Pa shear stress for 30 min [**(C)** and **(D)** shear-treated erythrocyte deformability]. These deformability curves present the shear stress-elongation index (EI) curves for 10 consecutive shear stresses: 0.3, 0.54, 0.96, 1.73, 3.1, 5.56, 9.97, 17.88, 32.07, and 57.50 Pa. Pre, pre-intervention; Post, post-intervention. Values are mean ± SD. ^*^ *p* < 0.05, pre or post.

**Figure 3:**
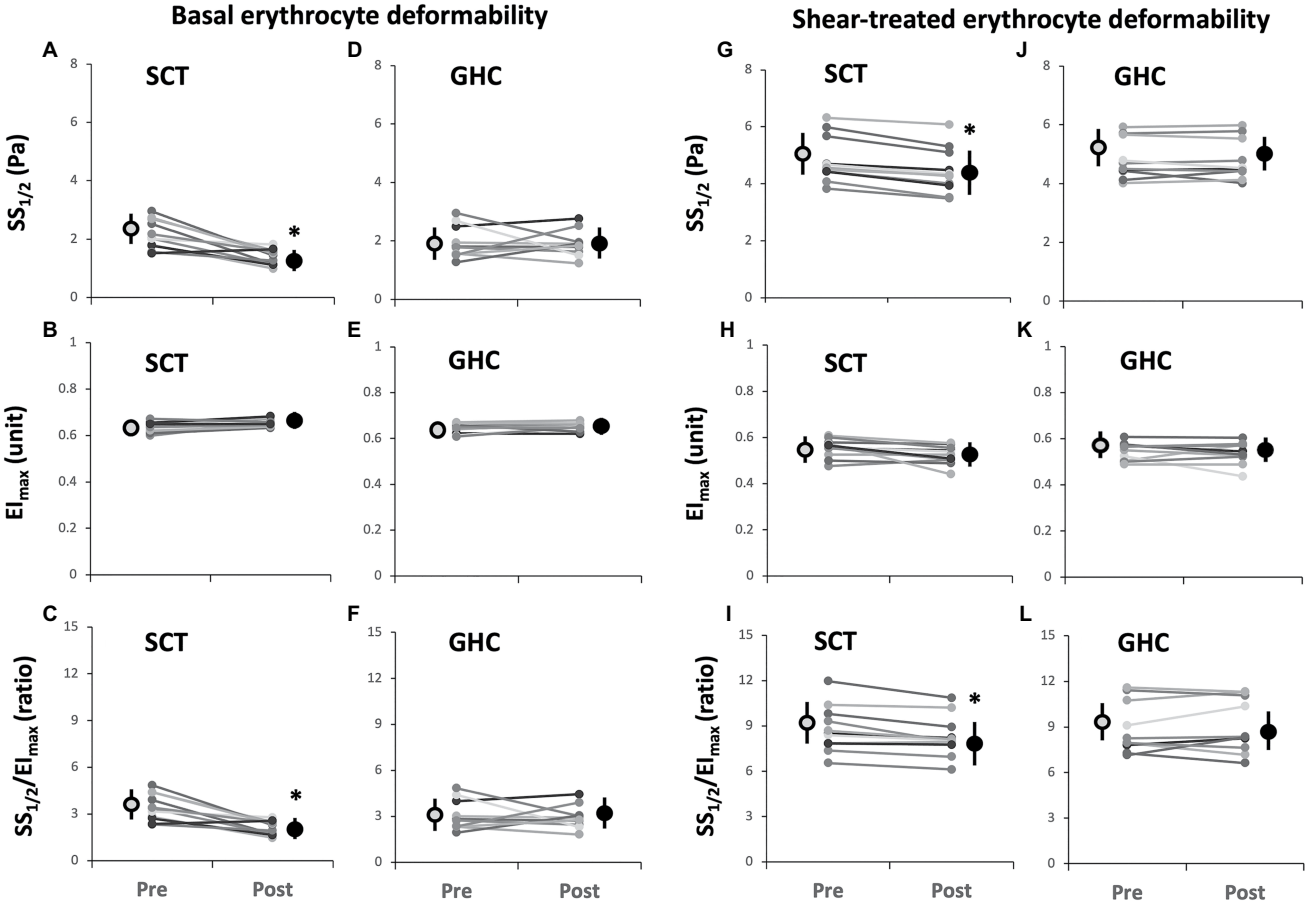
Effect of SCT and GHC on basal [**(A-F)**] and shear-treated [**(G-L)**] erythrocyte deformability in PAD. SS_1/2_, shear stress at 50% maximal elongation defined as semi-maximal shear stress; EI_max_, maximal deformability; and SS_1/2_/EI_max_, ratio of SS_1/2_ to EI_max_. Pre, pre-intervention; Post, post-intervention. Values are mean ± SD. ^*^ *p* < 0.05, pre or post.

Supervised cycling training for 12 weeks significantly increased basal (Figure 2A) and shear stress-treated (Figure 2C) erythrocyte membrane deformability as compared with unchanged values in GHC for 12 weeks (Figures 2B,D) at shear stress from 1 to 10 Pa during membrane deformability test. The exercise regimen also decreased basal SS_1/2_ (Figures 3A, *p* < 0.05) and ratio of SS_1/2_ to EI_max_ (Figure 3C, *p* < 0.05) but not the EI_max_ (Figure 3B). GHC did not decrease the above measurements (Figures 3D–F). A significant decrease (*p* < 0.05) of erythrocyte membrane deformability was observed in all participants after high shear stress (50 Pa) treatment for 30 min (Figures 3G–L). Similar expressions of significantly decreased SS_1/2_ (*p* < 0.05) after SCT (Figures 3G–I) and non-significant changes of SS_1/2_ after GHC (Figures 3J–L) were observed in high shear stress-treated erythrocytes.

## Erythrocyte Osmotic Deformability

**Figure 4:**
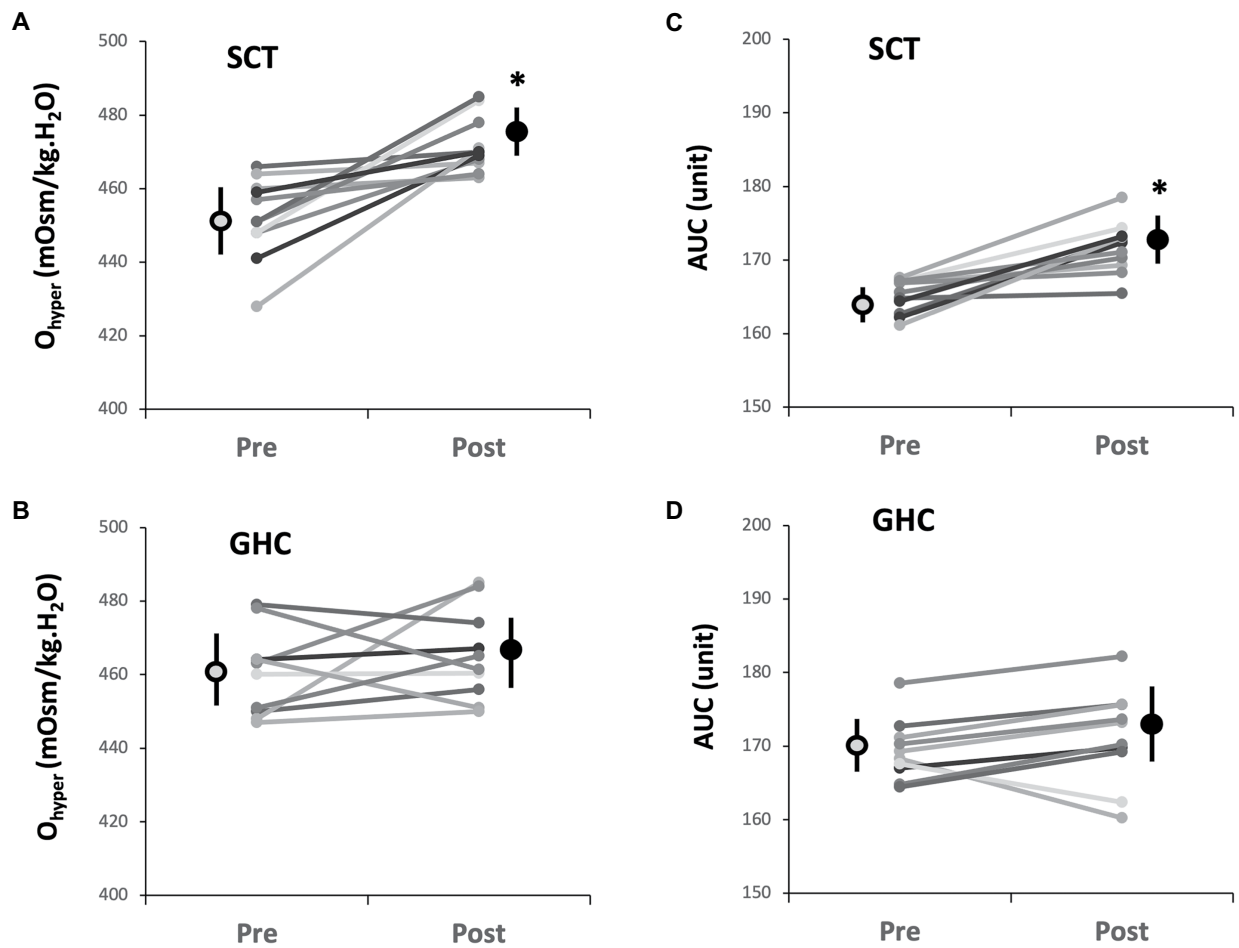
Effect of SCT and GHC on erythrocyte osmotic deformability [**(A)** and **(B)** O_hyper_; **(C)** and **(D)** AUC] in PAD. O_hyper_, osmolality in the hypertonic region corresponding to 50% maximal elongation index (EI_max_); AUC, area under the individual EI-osmolarity curve. Pre, pre-intervention; Post, post-intervention. Values are mean ± SD. ^*^ *p* < 0.05, pre or post.

Supervised cycling training for 12 weeks promoted O_hyper_ (Table 3 and Figure 4A, *p* < 0.05) and it is AUC (Table 3 and Figure 4C, *p* < 0.05) but did not change EI_max_, EI_min_, EI_hyper_, O_max_, or Q_min_ on erythrocytes (Table 3). However, 12-week GHC produced no change in these parameters of erythrocyte osmotic deformability (Table 3, Figures 4B,D).

## Discussion

Although supervised exercise program in addition to pharmacological therapy is recommended to increase exercise tolerance in PAD individuals (Kithcart and Beckman, 2018), optimal exercise dosage was not described in current guidelines. Therefore, study of 36 sessions of supervised cycling training at the first VT for individuals suffered from PAD was performed. Notably, this study is the first to demonstrate that the exercise regimen effectively improved erythrocyte membrane stability and osmotic deformability of erythrocytes in individuals with PAD. The increased erythrocyte deformability enhances peripheral tissue O_2_ delivery, which may promote peripheral tissue O_2_ extraction and contribute to the increase of O_2_ consumption during exercise based on the Fick equation (Malek and Coburn, 2008). Our findings support the supervised exercise program improved functional aerobic capacity and health-related QoL in individuals with PAD.

The exercise intervention enhanced the aerobic capacity of PAD individuals to independently achieve activities of daily living, thus improving their QoL (Treat-Jacobson et al., 2019a,b). In this investigation, 12 week of SCT at the first VT increased $\dot{\text{V}}$O_2peak_ by about 25% as well as 6MWT distances by about 25% (terminal distance) to 50% (pain-free distance), and decreased $\dot{\text{V}}$_E_-$\dot{\text{V}}$CO_2_ slope. The improvement of $\dot{\text{V}}$O_2peak_ in our exercise regimen was better than the 16% increase of $\dot{\text{V}}$O_2peak_ in those underwent revascularization therapy (Barkat et al., 2021) and the 13% increase of $\dot{\text{V}}$O_2peak_ in those underwent 12-week of pain-free walking on treadmill (Hiatt et al., 1994). These clinical observations imply that higher exercise intensity may be beneficial in cardiorespiratory fitness of PAD patients. The increase of 6MWT distance in the present work was similar as the outcomes of the Study to Improve Leg Circulation (SILC) (McDermott et al., 2009) and the Group Oriented Arterial Leg Study (GOALS) trials (McDermott et al., 2013). These physiological adaptations could finally promote physical and mental QoL in our observations.

The ventilatory parameters obtained from the graded exercise test may convey information regarding the prognosis of circulatory disorders (Arena et al., 2007). $\dot{\text{V}}$O_2peak_ is an indicator of systemic aerobic capacity, whereas $\dot{\text{V}}$_E_-$\dot{\text{V}}$CO_2_ slope commonly assesses ventilatory efficiency and is a dominant predictor of survival in people with circulatory disorders (Arena et al., 2007). Additionally, the 6MWT distance provides information regarding exercise endurance, which responds to therapy and prognosis across a broad range of chronic cardiopulmonary disorders (ATS Committee on Proficiency Standards for Clinical Pulmonary Function Laboratories, 2002). The supervised exercise training may effectively enhance the ability of PAD patients to cope with the physical demands of daily activity and thus improve their psychosocial state. Furthermore, the better health-related QoL produced by SCT may be associated with less potential for mortality in people with PAD and hence reduce the financial burden on their healthcare system (Treat-Jacobson et al., 2019a,b).

The essential function of erythrocytes is to deliver O_2_ to tissues by being highly deformable in order to pass through capillaries in the microcirculation (Baskurt and Meiselman, 2003; Barshtein et al., 2007). Erythrocyte deformability modulated by shear stress serves as a compensating mechanism for maintaining adequate microcirculatory perfusion (Saldanha and de Almeida, 2011). However, shear stress has a biphasic effect on the mechanical properties of erythrocytes depending on the duration and magnitude of the applied shear stress (Nevaril et al., 1968; Meram et al., 2013). Exposure to prolonged shear stress within the physiological range improves erythrocyte deformability (Meram et al., 2013), whereas exposure to pathologically high shear stress results in mechanical damage of erythrocytes (Nevaril et al., 1968). It has been known that shear stress >30 pa could impair erythrocyte deformability (Simmonds and Meiselman, 2017). In this investigation, pre-treatment at pathological high shear stress (50 Pa for 30 min) considerably increased the ratio of SS_1/2_ to EI_max_ on erythrocytes, as reflected by the decreased deformability of erythrocytes undergoing pathological shear force. The experimental results provide a model that may be used to predict the change in erythrocyte deformability following exposure to a pathological shear stress, which resembles the flow condition in stenotic arteries.

A previous investigation demonstrated that high-intensity interval training improved aerobic capacity and efficiency by depressing aggregability and enhancing deformability of erythrocytes in patients with heart failure (Wang et al., 2013). In this study, SCT, rather than GHC, enhanced basal erythrocyte deformability and erythrocyte membrane stability undergoing high shear stress, as indicated by a decrease of the shear-treated erythrocyte SS_1/2_/EI_max_ ratio. Findings of improved erythrocyte membrane stability after cycling training at the first VT in the present work were similar as cycling training at the alternating intensity of 40 and 80% $\dot{\text{V}}$O_2_R in our previous study using healthy sedentary males (Huang et al., 2019). With respect to underlying mechanisms of the SCT effects on erythrocyte rheological functions remain unclear, further investigations need to be undertaken. Nitric oxide (NO) derived from erythrocytes may regulate the deformability of erythrocyte membranes under shear flow (Bor-Kucukatay et al., 2003).

S-nitrosylation of cytoskeletal proteins, most likely α- and β-spectrins, caused by NO is involved in the active regulation of erythrocyte deformability (Grau et al., 2013). A previous study further demonstrated that moderate-intensity exercise increased vascular shear stress to enhance erythrocyte NO synthase activity and NO production by activating the PI3-kinase/Akt kinase pathway, thereby improving erythrocyte deformability (Suhr et al., 2012). Our early studies also demonstrated that this exercise regimen elevated plasma NO metabolite levels and endothelium-dependent dilation in skin vasculature (Wang et al., 2002; Wang, 2005). Atherosclerosis, the pathology underlying PAD, is a chronic inflammatory disease of the artery wall initiated by elevated low-density lipoprotein (LDL) level (Saldanha et al., 2012). *In vitro* study, blood aliquots enriched with LDL or high-density lipoprotein (HDL) showed significant higher erythrocyte aggregation than untreated blood aliquots (Saldanha et al., 2012). However, *in vivo* study, increased HDL concentration stimulated the release of NO from vascular endothelium, consequently improving blood rheology that included depressed erythrocyte aggregation, enhanced erythrocyte deformability, and lowered blood viscosity (Moriarty and Gibson, 2005). Additionally, NO retards myeloperoxidase-associated lipid peroxidation by acting as an antioxidant (Osawa, 2018). Our recent study also indicated that SCT decreased plasma myeloperoxidase level in PAD (Lin et al., 2021) to protect erythrocytes against oxidative damage and consequently improved the rheological function in people with PAD (Koutakis et al., 2018). The present work supported that moderate-intensity exercise facilitated erythrocyte NO synthase activity and NO generation, thereby improving erythrocyte deformability under shear flow. However, lipoprotein profile characteristics in the study could not endorse the exercise benefit because of the pharmacological therapy.

Erythrocyte deformability is crucially affected by changes in cell shape and volume caused by osmotic stress (Baskurt and Meiselman, 2003). In the present investigation, increased erythrocyte O_hyper_ level caused by SCT may provide a rapid pathway for promoting water inflow and limiting water outflow at hypertonicity, thereby contributing to improved osmotic deformability of the erythrocyte in PAD patients. Aquaporin 1 (AQP1), a water transport channel, facilitates water transport across the erythrocyte membrane and is responsible for the rapid response of the cell volume to changes in plasma osmolality (Sugie et al., 2018). Moreover, AQP1 on RBC prevents osmotic loss of water under hypertonic condition (Sugie et al., 2018). Our previous investigation demonstrated an increase in erythrocyte AQP1 expression after high-intensity interval training on a bicycle ergometer, which may provide a rapid pathway for promoting water inflow and limiting water outflow at hypertonicity, thereby contributing to improved osmotic deformability of the erythrocyte (Huang et al., 2019). Additionally, the cycling training also effectively alleviated hypoxia-evoked erythrocyte osmotic fragility by improving band 3 function on erythrocytes (Chou et al., 2016). In the present investigation, SCT for 12 weeks augmented O_hyper_ and the AUC in erythrocyte osmotic deformability. Accordingly, SCT may enhance aquaporin-mediated erythrocyte susceptibility to osmotic stress in individuals with PAD.

## Conclusion

In the present study, 12 week of SCT at the first VT enhanced functional aerobic capacity and improved health-related QoL in individuals with PAD. This exercise regimen also improved erythrocyte membrane stability and osmotic deformability of erythrocytes. These experimental findings may facilitate the identification of an effective exercise regimen to increase physical performance and improve efficacy for hemorheological functions in people with PAD. Although these results regarding erythrocyte rheological parameters have high values of statistical power ranging from 0.843 to 1.000, the small sample size in each group is still a major limitation in the present work.

## Data Availability Statement

The original contributions presented in the study are included in the article/Supplementary Material, further inquiries can be directed to the corresponding author.

## Ethics Statement

The studies involving human participants were reviewed and approved by Chang Gung Medical Foundation, Institutional Review Board. The patients/participants provided their written informed consent to participate in this study.

## Author Contributions

C-CH, Y-TL, and J-SW conceived and designed the experiments. Y-TL, T-CF, and S-CH performed the experiments. C-CH, Y-TL, T-CF, S-CH, C-HL, and J-SW analyzed the data. C-CH and Y-TL drafted the original manuscript. J-SW reviewed and edited the manuscript. All authors critically revised the manuscript for important intellectual content and approved the final manuscript.

## Funding

The research was funded by the Ministry of Science and Technology, Taiwan (grant nos. 108-2314-B-182-039-MY3 and 107-2314-B-182A-165), the Linkou and Keelung Chang Gung Medical Research Program (grant nos. CMRPD1J0221 and CMRPG2F0193), and the Higher Education Sprout Project by the Ministry of Education in Taiwan (grant no. EMRPD1L0371) for pure academic interesting.

## Conflict of Interest

The authors declare that the research was conducted in the absence of any commercial or financial relationships that could be construed as a potential conflict of interest.

## Publisher’s Note

All claims expressed in this article are solely those of the authors and do not necessarily represent those of their affiliated organizations, or those of the publisher, the editors and the reviewers. Any product that may be evaluated in this article, or claim that may be made by its manufacturer, is not guaranteed or endorsed by the publisher.
