# Supplementary material for: Supervised Cycling Training Improves Erythrocyte Rheology in Individuals With Peripheral Arterial Disease
Source: Front Physiol. 2022 Jan 5;12:792398. doi: 10.3389/fphys.2021.792398 (PMC8766405; doi:10.3389/fphys.2021.792398)

Supplementary Materials

Figure 1: Analysis of erythrocyte osmotic deformability using the osmotic gradient ektacytometry. The device generated a constant shear stress of 30 Pa while continuously aspirating the sample into the measurement site and while changing the osmolarity of the medium using gradual osmolarity mixtures of polyvinylpyrrolidone solutions; therefore, the elongation index (EI) was continuously registered. O_{max}, osmolality at which EI is maximum (EI_{max}); O_{min}, osmolality at which EI is minimal (EI_{min}); O_{hyper}, osmolality in the hypertonic region corresponding to 50% EI_{max}; EI_{hyper}, EI in O_{hyper}; AUC, area under the individual EI–osmolarity curve; Pre-SCT, before supervised cycling training; Post-SCT, after supervised cycling training.

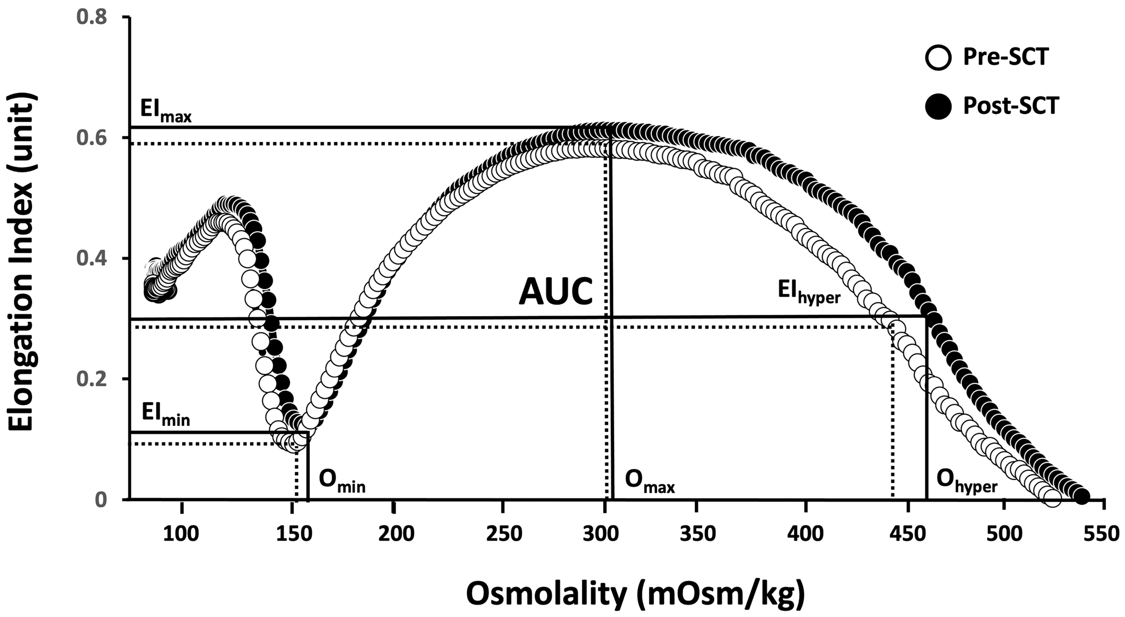

Supplement: Supplementary file 1 [file Data_Sheet_1.pdf]
